# Impact of NPS rates on growth and yield of garlic (*Allium sativum* L.) varieties in greenhouse

**DOI:** 10.1016/j.heliyon.2024.e39963

**Published:** 2024-10-29

**Authors:** Eshet Lakew Tesfaye, T. Bayih

**Affiliations:** Department of Biotechnology, College of Natural and Computational Sciences, Hawassa University, Hawassa, Ethiopia

**Keywords:** Garlic production, Garlic varieties, Growth, NPS, Yield parameters

## Abstract

Garlic (*Allium sativum* L.) has both culinary and medical applications. However, the low and uneven nutrient availability in the soil frequently limits the garlic yield. This study aimed to select adaptable garlic varieties under greenhouse conditions using a pot of soil taken from Awada and Wondo Genet and to identify the optimum rates of the newly introduced mineral nitrogen, phosphorus, and sulfur (NPS) fertilizer on the growth, yield, and yield parameters of the crop. Four garlic varieties; Bishoftu Nech, Tsedey 92, Kuriftu, and local variety were used and treated with four levels of NPS (0-0-0, 78.75-69-12.75, 105-92-17, and 131.25-115-21.25 kg ha^−1^). The experiment was triplicated and conducted with a randomized complete block design (RCBD). Growth characteristics of the four garlic varieties were evaluated, including days to 50 % emergence, plant height, leaf number per plant, leaf length, fresh weight, dry weight, average bulb weight, and total bulb yield. The findings of this study indicated that the growth, yield, and yield parameters of all four garlic varieties from Awada soil rose as the rates of NPS increased. However, all growth and production parameters of the four garlic varieties gradually fell, starting from the blanket recommendation from Wondo Genet soil. According to this research, the usage of NPS at the rate of (131.25-115-21.25 kg ha^−1^) resulted in improved growth in Awada soil. Similarly, the rate of (78.75-69-12.75 kg ha^−1^) was optimum at Wondo Genet soil. In conclusion, determining the optimum rates of the NPS is essential to increase garlic productivity.

## 1 Introduction

Garlic (*Allium sativum* L.) is one of the most cultivated and highly consumed vegetables in the world [1]. It is widely used as a spice in food and as medication in contemporary medicine to treat and cure various illnesses [2]. For more than 5000 years, the cultivation of garlic as a spice and medicinal plant has been recognized for its great significance [3]. A milder environment is needed in the early phases to promote vegetative growth, while greater temperatures are necessary for garlic throughout bulbing. It is generally agreed upon that the optimal temperature range for producing garlic is between 12 and 24 °C on average [4]. Garlic has a limited ability to collect nutrients due to its unbranched, shallow-rooted root system. The use of fertilizers is essential to increasing garlic bulb growth, output, and marketable proportions while maintaining bulb quality [1]. The application of mineral fertilizers in adequate quantities promotes improvements in leaf area and yield of garlic [5]. Garlic's growth in terms of plant height, number of leaves, neck diameter, and leaf area index was greatly affected by the application of inorganic N, P, and S fertilizers [6]. Similarly, garlic absorbs N and K more readily than Cl, whereas it absorbs P less readily [7].

Garlic is widely grown all over the world and is incredibly versatile. China, India, Bangladesh, Korea, Egypt, and Spain are the top producers worldwide [7]. Asia is where garlic is mostly farmed (87 %), with China and India together producing 78 % of the world's garlic [8]. With 1,546,741 ha of land, 28.49 million tons of garlic were produced worldwide in 2018. As per the FAOSTAT 2018 report, the overall production in Ethiopia was 12,429 ha of land yielding an average of 124,801 tons productivity of 10.04 t ha ^−1^, a remarkably low amount in comparison to 18.4 t ha ^−1^ on a global average [9].The quick nutrient loss on smallholder farms is one of the main issues influencing crop output in Africa, including Ethiopia [10]. Low nutrient availability and soil moisture stress are the primary issues limiting garlic output in many garlic-producing regions. This stress prevents nitrogen, phosphorus, and sulfur from being released from soil organic matter and from being absorbed by plant roots [11].

Garlic is one of the most important vegetable crops grown in Ethiopia, both under irrigation and with rain, but production is extremely low both nationally and regionally because of inadequate agronomic methods and improper fertilizer application, both in terms of rate and type [12]. The yield variations can be attributed more to crop management and technological resources than to the genetic composition and performance of the cultivars used [13]. Soils with a pH between 6.5 and 7.5, high organic matter content, good drainage, and the ability to hold onto enough moisture are ideal for growing garlic [14]. The lack of minerals and soil water in many Ethiopian regions that produce garlic was preventing growth and productivity from achieving their maximum potential [15]. Farmers have been using urea fertilizers and the nitrogen and phosphorus included in Diammonium Phosphate (DAP) to maximize plant yields. The Ethiopian Ministry of Agriculture reports that DAP has been replaced by the new compound fertilizer (NPS), which has the following ratios: 19 %, 38 %, and 7 % for nitrogen (N), phosphorus (P_2_O_5_), and sulfur (S) [4].

However, little is known about how NPS influences the development and productivity of garlic by Ethiopian researchers and farmers in the Sidama region. Furthermore, farmers do not have any improved variety; they only grow the local variety of garlic [16]. Understanding the effects of the recently released NPS fertilizer with sulfur and determining the optimal agronomic and economic threshold present even greater challenges for scientists and farmers [14]. Particularly for the garlic plant, balanced mineral fertilizer dosages increased output, photosynthetic productivity, and leaf area, which resulted in a notable increase in overall production [17]. Moreover, the variety, season, and type of soil all influence the nutrient requirements of the crop. Garlic variants of the “Tseday" are grown in many areas with a blanket recommendation rate of 105 kg N ha^−1^ and 92 kg P_2_O_5_ ha^−1^ [18]. Furthermore, this is utilized as a blanket recommendation for “Chelenko I" [19]. This suggests that there are no optimum fertilizer application rates that should be followed to produce the crop in the studied area. Because fertilizer is applied nationwide based on blanket recommendations rather than considering the soil fertility levels of specific places, which is frequently unprofitable [20].

Therefore, the purpose of this study was to determine the impact of four NPS fertilizer rates on the productivity and adaptability of the four garlic varieties and to select the adaptable variety and optimum rates of NPS fertilizer in the study area.

## 2 Materials and methods

### 2.1 Description of the study area

For this research, a greenhouse at the temperature of (24 °C ± 2 °C) and humidity of (70 %) located at the main campus of Hawassa University was used. The greenhouse was designed with a mid-sized, even-span form, allowing maximum exposure to sunlight and optimal airflow management utilizing plastic specifically polycarbonate as the primary covering material. Hawassa city is positioned along the shores of Lake Awassa in the Great Rift Valley. It is situated approximately 273 km south of Addis Ababa, 130 km east of Sodo, and 75 km north of Dilla. The city serves as the central hub for the Sidama region. Positioned on the Trans-African Highway, connecting Cairo and Cape Town, it sits at an altitude of 1708 m (5604 feet) above sea level, at latitude and longitude coordinates 7°3′N 38°28′E. The term “Hawassa" originates from a Sidamic expression meaning “wide body of water".

### 2.2 Description of the plant materials

The experiment involved utilizing three improved garlic types (Bishoftu Nech, Tsedey 92, and Kuriftu), all of which were developed and released by the Debre Zeit Agricultural Research Center, along with one local variety. The four garlic varieties will be evaluated for their yield and yield-related parameters namely; Days to 50 % emergence (d), Plant height (cm), Leaf number per plant (n), Leaf length (cm), Fresh weight (g), Dry weight (g), Average bulb weight (g) and Total bulb yield (t ha^−1^).

### 2.3 Soil analysis

Before conducting experiments, samples of topsoil were examined physiochemically. The pH of the soil was measured using a 1:2.5 soil ratio of water suspension method and a pH meter. The wet digestion procedure yielded the amount of organic matter (OM) [21] **and** the percentage of soil organic matter (OM) was divided by1.724 to determine the percentage of organic carbon (OC), working under the premise that OM is mostly made up of 58 % carbon [22]**. Total nitrogen was analyzed using the Kjeldahl method** [23]**.** Using a spectrophotometer with an 880 nm wavelength, the available (P) in the soil was extracted using the Bray-II method, employing a colorimetric method with an ammonium mixture, sulfuric acid, potassium antimony, and molybdate, with tartrate as an indicator [24]. A flame photometer was used to assess the amount of exchangeable basic (K) ions extracted using a 1 M ammonium acetate (NH₄C₂H₃O₂) solution at pH 7 [24]. Dry ashing [25] and sulfate detection using ion chromatography [26] were used to estimate total sulfur.

#### 2.3.1 Soil physicochemical properties

Overall, although there are some variations in the values of the majority of the physicochemical attributes of the soil between the Awada and Wondo Genet soil types, the majority of these variations are not statistically significant (Table 1).

**Table 1:** Physicochemical properties of Awada and Wondo Genet soils at the depth of 0–40 cm.

Parameters	Value	
	Awada	Wondo Genet
P (ppm)	18.3	17.1
pH	5.6	6.4
OM (%)	5.2	5.8
OC (%)	3.2	3.8
N (%)	0.4	0.6
K (ppm)	285.4	310.5
S (ppm)	12.3	9.8
C: N ratio	10.1	9.6

Note: P = phosphorus; OM = organic matter; OC = organic carbon; N = nitrogen; pH = power of hydrogen; ppm = parts per million; C: N = carbon: nitrogen; % = percentage; S= Sulpher; K= Potassium.

### 2.4 Experimental design and Layout

**Fig. 1:**
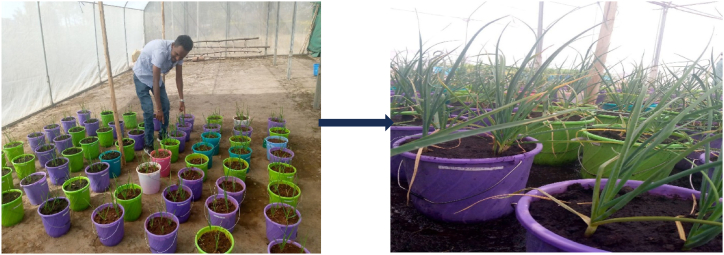
Garlic plantation in the greenhouse condition.

A greenhouse-based pot experiment was conducted (Fig. 1), following the methods outlined in Ref. [27], with certain modifications. The experiment was conducted at a temperature of 26 °C, employing four rates of NPS fertilizer. Sixteen treatment combinations, with three replicates each (totaling 96 randomized pots, including the control), were applied. Each pot measuring 25 cm × 20 cm × 16 cm contained 4 kg of soil and was planted with four garlic cloves. Soil moisture was maintained at 70 % of its water-holding capacity through daily watering. NPS containing 19 % N, 38 % P_2_O_5_, and 7 % S was used as a source of fertilizer.

### 2.5 Experimental treatment detail

Four different rates of NPS, including the control, were applied in the treatments, following the guidelines outlined in Ref. [12]. The treatments were conducted at 25 % below the blanket recommendation (78.75-69-12.75 kg ha^−1^), blanket recommendation (105-92-17 kg ha^−1^), 25 % above the blanket recommendation (131.25-115-21.25 kg ha^−1^), and the control (0-0-0 kg ha^−1^). The experiment followed a RCBD with three replications. Thirty days after planting, half of the aforementioned NPS rates were applied.

### 2.6 Experimental procedures

At both the Awada and Wondo Genet campuses of Hawassa University, a 5 × 5 m plot of land was selected and cleared of any litter and plant remnants. Soil samples were collected using a soil auger, reaching a depth of 0–40 cm, and manually broken up to remove any clods. Before the treatments were applied, the soil samples were air-dried, sieved through a 5 mm sieve, and analyzed. The experimental pots were then filled with the resulting fine soil.

Cloves were selected from bulbs based on size categories, including large (2.0–2.5 g), medium (1.5–1.9 g), and small (1.0–1.49 g), as described in Ref. [28]. Planting utilized cloves falling within the large to medium size range (2.5–1.5 g) from these categories. These cloves were manually inserted using a hand into the soil in the pots at a depth of 3–4 cm. Harvesting was conducted by hand when approximately 70 % of the leaves had fallen, as noted in Ref. [29]. The harvested bulbs were sun-dried for 10 days and covered with leaves to prevent sunburn, as per [29]. After a week of drying, trimming of the necks and roots was performed.

### 2.7 Yield parameters for garlic production

The yield parameters for garlic production for the study area were evaluated following the procedures in Ref. [4].

#### 2.7.1 Days to 50 % emergence (d)

It was determined by counting when about 50 % of the plants emerged.

#### 2.7.2 Plant height (cm)

It was determined by measuring the height of four plants using a ruler from the pot and taking the average value.

#### 2.7.3 Leaf number per plant (n)

It was computed by dividing the total number of leaves counted from the sampled plants by the number of sampled plants to get the mean leaf number per plant. It is the mean number of leaves produced by the sampled plants.

#### 2.7.4 Leaf length (cm)

The average length of the longest leaf, at an active leaf growth stage, was measured in centimeters from the four plants in the pot.

#### 2.7.5 Fresh weight (g)

Fresh weight was measured from four garlic plants from each plot by using a sensitive balance.

#### 2.7.6 Dry weight (g)

After being dried from four plants for 48 h at a temperature of 70 °C in an oven, the dry weight of the above-ground biomass was measured in grams. Above-ground dry biological yield per plant was determined using averages.

#### 2.7.7 Average bulb weight (g)

Four bulbs were removed from the pot and weighed using a delicate balance to get the measurement. Bulb weight was used to represent the average weight.

#### 2.7.8 Total bulb yield (t ha ^−1^)

To calculate the yield, the weight of all collected bulbs (g/pot) in the pot (0.38 m^2^) was converted to t ha ^−1^.

### 2.8 Data analysis

For each treatment, the mean value of the above-described parameters was computed and subjected to analysis of variance (ANOVA) using SPSS computer software version 26 with a general linear model procedure. The mean separation test was done using List Significant Difference (LSD) followed by Duncan's Multiple Range Test (DMRT) test at a 5 % probability level.

## 3 Results

### 3.1 Days to 50 % emergence (d)

Days to 50 % emergence were significantly affected (*P* < 0.05) by the different rates of NPS fertilizer. The local variety from Awada soil, which was fertilized at a rate of (131.25-115-21.25 kg ha^−1^) NPS, emerged four days after planting, while Kuriftu showed delayed emergence at the same time that the local and Kuriftu varieties from Wondo Genet soil, which were fertilized at a rate of (131.25-115-21.25 kg ha^−1^) NPS, emerged six days after planting (Table 2).

**Table 2:** Effect of varying rates of NPS on days to 50 % emergence (d).

Rate of NPS (kg ha^−1^)	Mean of each garlic variety from Awada soil				Mean of each garlic variety from Wondo Genet soil			
	Bishoftu Nech	Tsedey 92	Kuriftu	local	Bishoftu Nech	Tsedey 92	Kuriftu	local
0-0-0 (control)	10.33^a^	11.00^a^	11.67^a^	10.33^a^	12.00^a^	11.67^a^	10.00^a^	10.67^a^
78.75-69-12.75	7.00^b^	6.00^b^	8.00^b^	4.33^b^	7.00^b^	10.00^b^	6.67^b^	8.00^b^
105-92-17	6.67^c^	5.33^c^	7.00^c^	4.00^c^	6.00^c^	8.33^c^	6.00^c^	7.00^c^
131.25-115-21.25	5.33^d^	4.33^d^	6.00^d^	4.00^c^	5.00^d^	7.00^d^	6.00^c^	6.00^d^
LSD (5 %) = 0.47; CV (%) = 32.7								

Means represented with the same letter(s) in columns and rows are not significantly different from each other. LSD (5 %) = least significant difference at *P* < 0.05; CV (%) = coefficient of variation; NPS = Nitrogen, Phosphors, and Sulfur; kg ha^−1^ = kilogram per hectare.

### 3.2 Plant height (cm)

Plant height was highly significantly (*P* < 0.05) affected by the different NPS rates. The highest and lowest mean plant height of Awada soil was (75.13 and 23.47 cm) by the application of (131.25-115-21.25 kg ha^−1^) and the control respectively. Whereas, it was (48.63 and 26.00 cm) by the application of (78.75-69-12.75 kg ha^−1^) and the control rates respectively in Wondo Genet soil (Table 3).

**Table 3:** Effect of varying rates of NPS on plant height (cm).

Rate of NPS (kg ha^−1^)	Mean of each garlic variety from Awada soil				Mean of each garlic variety from Wondo Genet soil			
	Bishoftu Nech	Tsedey 92	Kuriftu	local	Bishoftu Nech	Tsedey 92	Kuriftu	local
0-0-0 (control)	30.13^d^	28.20^d^	27.77^d^	23.47^d^	35.20^d^	32.40^d^	30.10^d^	26.00^d^
78.75-69-12.75	39.90^c^	46.07^c^	38.63^c^	32.77^c^	43.60^a^	42.33^a^	48.63^a^	33.83^a^
105-92-17	45.50^b^	57.40^b^	40.00^b^	33.20^b^	38.97^b^	38.23^b^	40.20^b^	30.37^b^
131.25-115-21.25	55.40^a^	75.13^a^	41.43^a^	38.30^a^	37.63^c^	37.13^c^	39.10^c^	30.63^c^
LSD (5 %) = 0.89; CV (%) = 26.3								

Means represented with the same letter(s) in columns and rows are not significantly different from each other. LSD (5 %) = least significant difference at *P* < 0.05; CV (%) = coefficient of variation; NPS = Nitrogen, Phosphors, and Sulfur; kg ha^−1^ = kilogram per hectare.

### 3.3 Leaf number per plant (n)

Analysis of variance indicated that rates of NPS fertilizer significantly (*P* < 0.05) affected the leaf number per plant. The highest and the lowest leaf numbers were recorded as (12.60 and 6.57) by the application of (131.25-115-21.25 kg ha^−1^) and the control rates respectively in Awada soil. Whereas, the highest and the lowest leaf numbers were recorded as (14.43 and 3.90) by the application of (78.75-69-12.75 kg ha^−1^) and the control rates from Wondo Genet soil respectively (Table 4).

**Table 4:** Effect of varying rates of NPS on leaf number per plant (n).

Rate of NPS (kg ha^−1^)	Mean of each garlic variety from Awada soil				Mean of each garlic variety from Wondo Genet soil			
	Bishoftu Nech	Tsedey 92	Kuriftu	local	Bishoftu Nech	Tsedey 92	Kuriftu	local
0-0-0 (control)	6.87^d^	9.00^d^	7.17^d^	6.57^d^	4.37^d^	3.90^d^	7.50^d^	4.23^d^
78.75-69-12.75	8.83^c^	9.57^c^	7.73^c^	7.77^c^	11.93^a^	10.97^a^	14.43^a^	7.87^a^
105-92-17	9.23^b^	10.50^b^	8.27^b^	8.83^b^	9.93^b^	10.10^b^	12.10^b^	6.43^b^
131.25-115-21.25	11.50^a^	12.60^a^	11.53^a^	9.93^a^	8.00^c^	8.80^c^	11.37^c^	6.03^c^
LSD (5 %) = 0.56; CV (%) = 28.5								

Means represented with the same letter(s) in columns and rows are not significantly different from each other. LSD (5 %) = least significant difference at *P* < 0.05; CV (%) = coefficient of variation; NPS = Nitrogen, Phosphors, and Sulfur; kg ha^−1^ = kilogram per hectare.

### 3.4 Leaf length (cm)

Leaf length was highly influenced by the application of the different rates of NPS fertilizer (*P* < 0.05). The highest and lowest leaf lengths (40.20 and 23.03 cm) were recorded by the application of (131.25-115-21.25 kg ha^−1^) and the control rates from Awada soil respectively. Whereas, the highest and lowest leaf lengths (28.63 and 16.93 cm) were recorded by the application of (78.75-69-12.75 kg ha^−1^) and the control rates from Wondo Genet soil respectively (Table 5).

**Table 5:** Effect of varying rates of NPS on leaf length (cm).

Rate of NPS (kg ha^−1^)	Mean of each garlic variety from Awada soil				Mean of each garlic variety from Wondo Genet soil			
	Bishoftu Nech	Tsedey 92	Kuriftu	local	Bishoftu Nech	Tsedey 92	Kuriftu	local
0-0-0 (control)	23.47^d^	23.97^d^	23.13^d^	23.03^d^	20.23^d^	20.17^d^	20.87^d^	16.93^d^
78.75-69-12.75	25.40^c^	28.63^c^	27.03^c^	26.03^c^	26.80^a^	25.23^a^	28.63^a^	22.00^a^
105-92-17	27.37^b^	31.27^b^	28.53^b^	27.20^b^	25.06^b^	22.40^b^	26.90^b^	19.80^b^
131.25-115-21.25	35.77^a^	40.20^a^	32.70^a^	27.87^a^	23.17^c^	21.40^c^	25.87^c^	18.70^c^
LSD (5 %) = 0.99; CV (%) = 19.3								

Means represented with the same letter(s) in columns and rows are not significantly different from each other. LSD (5 %) = least significant difference at *P* < 0.05; CV (%) = coefficient of variation; NPS = Nitrogen, Phosphors, and Sulfur; kg ha^−1^ = kilogram per hectare.

### 3.5 Fresh weight (g)

The fresh weight of garlic was significantly (*P* < 0.05) affected by the different rates of NPS. The highest and lowest fresh weight was recorded (9.63 and 2.57 g) by the application of (131.25-115-21.25 kg ha^−1^) and the control from Awada soil respectively. On the other hand, the highest and lowest fresh weight (12.77 and 5.30 g) was recorded by the application of (78.75-69-12.75 kg ha^−1^) and the control rates from Wondo Genet soil respectively (Table 6).

**Table 6:** Effect of varying rates of NPS on Fresh weight (g).

Rate of NPS (kg ha^−1^)	Mean of each garlic variety from Awada soil				Mean of each garlic variety from Wondo Genet soil			
	Bishoftu Nech	Tsedey 92	Kuriftu	local	Bishoftu Nech	Tsedey 92	Kuriftu	local
0-0-0 (control)	3.53^d^	4.2^d^	4.33^d^	2.57^d^	6.67^d^	5.7^d^	6.03^d^	5.30^d^
78.75-69-12.75	4.27^c^	4.87^c^	5.50^c^	3.50^c^	11.13^a^	10.80^a^	12.77^a^	9.27^a^
105-92-17	6.33^b^	5.90^b^	6.47^b^	3.93^b^	9.00^b^	9.47^b^	11.23^b^	7.80^b^
131.25-115-21.25	7.53^a^	9.63^a^	8.27^a^	6.50^a^	8.07^c^	8.33^c^	8.67^c^	7.53^c^
LSD (5 %) = 0.29; CV (%) = 36.2								

Means represented with the same letter(s) in columns and rows are not significantly different from each other. LSD (5 %) = least significant difference at *P* < 0.05; CV (%) = coefficient of variation; NPS = Nitrogen, Phosphors, and Sulfur; kg ha^−1^ = kilogram per hectare.

### 3.6 Dry weight (g)

The dry weight of garlic was highly significantly (*P* < 0.05) affected by varying rates of NPS. The highest and lowest dry weight (6.03 and 2.03 g) was recorded by the application of (131.25-115-21.25 kg ha^−1^) and the control rates from Awada soil respectively. Whereas, the highest and lowest dry weight (5.83 and 2.13 g) was recorded by the application of (78.75-69-12.75 kg ha^−1^) and the control NPS rates from Wondo Genet soil respectively (Table 7).

**Table 7:** Effect of varying rates of NPS on dry weight (g).

Rate of NPS (kg ha^−1^)	Mean of each garlic variety from Awada soil				Mean of each garlic variety from Wondo Genet soil			
	Bishoftu Nech	Tsedey 92	Kuriftu	local	Bishoftu Nech	Tsedey 92	Kuriftu	local
0-0-0 (control)	2.50^d^	3.07^d^	2.17^d^	2.03^d^	2.13^d^	2.13^d^	2.63^d^	2.13^d^
78.75-69-12.75	2.93^c^	4.07^c^	3.70^c^	2.67^c^	4.40^a^	3.57^a^	5.83^a^	3.20^a^
105-92-17	4.10^b^	4.8^b^	4.23^b^	3.03^b^	3.90^b^	3.07^b^	4.47^b^	2.80^b^
131.25-115-21.25	4.93^a^	6.03^a^	4.70^a^	4.13^a^	3.07^c^	2.77^c^	3.43^c^	2.43^c^
LSD (5 %) = 0.56; CV (%) = 31.0								

Means represented with the same letter(s) in columns and rows are not significantly different from each other. LSD (5 %) = least significant difference at *P* < 0.05; CV (%) = coefficient of variation; NPS = Nitrogen, Phosphors, and Sulfur; kg ha^−1^ = kilogram per hectare.

### 3.7 Average bulb weight (g)

Analysis of variance showed that average bulb weight was significantly affected by NPS (*P* < 0.05). The highest and lowest average bulb weight (53.43 and 32.43 g) was recorded by the application of (131.25-115-21.25 kg ha^−1^) and the control from Awada soil respectively. Whereas, the highest and lowest average bulb weight (48.67 and 28.63 g) was recorded by the application of (78.75-69-12.75 kg ha^−1^) and the control from Wondo Genet soil respectively (Table 8).

**Table 8:** Effect of varying rates of NPS on average bulb weight (g).

Rate of NPS (kg ha^−1^)	Mean of each garlic variety from Awada soil				Mean of each garlic variety from Wondo Genet soil			
	Bishoftu Nech	Tsedey 92	Kuriftu	local	Bishoftu Nech	Tsedey 92	Kuriftu	local
0-0-0 (control)	33.07^d^	38.20^d^	35.40^d^	32.43^d^	37.23^d^	35.67^d^	40.97^d^	28.63^d^
78.75-69-12.75	41.93^c^	43.33^c^	43.47^c^	43.13^c^	45.73^a^	43.80^a^	48.67^a^	37.20^a^
105-92-17	43.93^b^	46.70^b^	46.07^b^	44.63^b^	42.37^b^	42.17^b^	45.67^b^	35.33^b^
131.25-115-21.25	47.40^a^	53.43^a^	48.63^a^	47.33^a^	40.73^c^	39.53^c^	43.23^c^	32.80^c^
LSD (5 %) = 0.72; CV (%) = 13.5								

Means represented with the same letter(s) in columns and rows are not significantly different from each other. LSD (5 %) = least significant difference at *P* < 0.05; CV (%) = coefficient of variation; NPS = Nitrogen, Phosphors, and Sulfur; kg ha^−1^ = kilogram per hectare.

### 3.8 Total bulb yield (t ha ^−1^)

Garlic variety total bulb yield was significantly affected by the different rates of NPS (*P* < 0.05). The highest and lowest bulb yield (16.83 and 6.37 t ha ^−1^) was recorded by the application of (131.25-115-21.25 kg ha^−1^) and the control rates from Awada soil respectively. Whereas, the highest and lowest bulb yield (15.53 and 4.40 t ha ^−1^) was recorded by the application of (78.75-69-12.75 kg ha^−1^) and the control rates from Wondo Genet soil respectively (Table 9).

**Table 9:** Effect of varying rates of NPS on total bulb yield (t ha ^−1^).

Rate of NPS (kg ha^−1^)	Mean of each garlic variety from Awada soil				Mean of each garlic variety from Wondo Genet soil			
	Bishoftu Nech	Tsedey 92	Kuriftu	local	Bishoftu Nech	Tsedey 92	Kuriftu	local
0-0-0 (control)	9.67^d^	10.03^d^	8.23^d^	6.37^d^	6.73^d^	5.73^d^	7.90^d^	4.40^d^
78.75-69-12.75	10.97^c^	12.70^c^	10.60^c^	8.43^c^	12.40^a^	10.73^a^	15.53^a^	9.63^a^
105-92-17	11.97^b^	14.57^b^	11.87^b^	9.03^b^	9.77^b^	9.67^b^	12.77^b^	8.87^b^
131.25-115-21.25	14.20^a^	16.83^a^	13.60^a^	10.67^a^	8.27^c^	7.80^c^	10.63^c^	7.30^c^
LSD (5 %) = 0.43; CV (%) = 28.1								

Means represented with the same letter(s) in columns and rows are not significantly different from each other. LSD (5 %) = least significant difference at *P* < 0.05; CV (%) = coefficient of variation; NPS = Nitrogen, Phosphors, and Sulfur; kg ha^−1^ = kilogram per hectare.

## 4 Discussion

**The results of this study indicated that utilizing NPS during garlic production positively influenced the growth and yield attributes of the selected garlic varieties. Adequate nutrient content in both the soil and crops is essential for achieving satisfactory garlic yields and maintaining quality.** However, this study faced a lack of access to the individual nutrients and advanced laboratory settings to study the detailed parameters including the molecular characterization of the garlic gene responsible for the maximum yield for sustainable garlic production and future garlic breeding programs.

The impact of various rates of NPS on the time taken for 50 % emergence significantly varied among the garlic varieties, ranging from four to six days (Table 2). Previous studies have reported earlier emergence (nine days) when using NPS at a rate of (105: 92:17 kg ha^−1^) [12]**.** This outcome validates that employing the highest rates of NPS accelerates the emergence of garlic bulbs. This acceleration could be attributed to the impact of accessible nitrogen, phosphorus, and sulfur on the initiation and growth of roots, thereby promoting early shoot emergence. The presence of these nutrients likely enhances the concentration of soluble carbohydrates, subsequently altering the distribution patterns of non-structural carbohydrates. These conditions collectively contribute to the earlier emergence of shoots [4].

Plant height, one of the yield parameters assessed across the four NPS rates, displayed a notable trend. The findings demonstrated a decrease in plant height with increasing rates of NPS, starting from the blanket recommendation from Wondo Genet soil (Table 3). The garlic plants exhibited accelerated growth when subjected to higher rates of NPS. This acceleration might be attributed to NPS aiding in metabolic activities, leading to the synthesis of coenzymes, phospholipids, and nucleic acids [4]**.** Among the treatments, applying 60 kg ha^−1^ of sulfur to the garlic led to the highest plant height of garlic plants [30]**.**

The study evaluated the number of leaves per plant across four garlic varieties by applying four rates of NPS. Results showed that as the rates of NPS increased, so did the number of leaves per plant. However, a decrease in leaf count was observed starting from the blanket recommendation from Wondo Genet soil (Table 4). These findings align with previous research, which also observed that the application of blended NPS promoted the growth and development of garlic, including increasing the number of leaves [31]. Increased levels of NPS could potentially lead to a greater number of leaves since nitrogen, phosphorus, and sulfur are crucial nutrients required for metabolic processes [4]. Increased application of NPS may boost leaf number due to increased nitrogen availability, which stimulates cell division and elongation, thereby promoting the growth of more leaves [32]. Furthermore, it enhanced protein synthesis, leading to increased accumulation of carbohydrates and other plant growth traits [33].

The experiment's outcomes revealed a significant impact of applying varied rates of NPS on leaf length. It was observed that as the rates of NPS increased, the length of leaves also increased across all garlic varieties grown from Awada soil. Furthermore, there was a decline in leaf length for all garlic varieties, starting from the blanket recommendation of different NPS rates from Wondo Genet soil (Table 5). The longer length of garlic leaves may be attributed to enhanced nutritional availability, particularly nitrogen (N), which fosters leaf elongation through the stimulation of cell division and enlargement, thus promoting overall plant growth [34]. This result aligns with the conclusions drawn by other researchers, who verified that the length of garlic shoots increased alongside higher levels of NPK [8].

The application of four rates of NPS had an impact on the fresh weight of the garlic varieties. The study's results demonstrated that a higher rate of NPS led to an increase in fresh weight for all varieties grown from Awada soil. Conversely, the findings also indicated that a higher rate of NPS, starting from the blanket recommendation, resulted in a decrease in fresh weight for all garlic varieties from Wondo Genet soil (Table 6). One potential explanation for the increase in fresh weight with increased NPS rates is the enhanced availability of nutrients in the root zone of the garlic plants [35]. On the contrary, an excessive increase in the application of NPS became unusable by the plant and might induce toxic effects on its growth [12].

The dry weight of garlic varieties in both soil types was significantly influenced by the application of four rates of NPS. Moreover, the dry weight of all garlic varieties increased proportionally with the application rate of NPS. However, further increases in NPS rates, starting from the blanket recommendation, resulted in reduced dry weight for each garlic variety (Table 7). This could be attributed to the impact of fertilizers containing high levels of phosphorus, sulfur, and nitrogen on the production of dry matter in garlic plants. Additionally, other researchers also observed that the treatment with phosphorus did not lead to a significant increase in garlic plant height [36]. Moreover, nitrogen nutrition did not produce any noticeable effects on the morphological traits of garlic [37].

The application of four rates of NPS significantly affected the average bulb weight of all garlic varieties in both soil types. Furthermore, the average bulb weights of all garlic varieties increased with higher rates of NPS. However, the dry weight of each garlic variety decreased with each additional increase in NPS rate, starting from the blanket recommendation (Table 8). Increased levels of sulfur might have enhanced the uptake of nitrogen, phosphorus, potassium, and sulfur by the crop, improving nutrient assimilation and allocation for bulb synthesis, thereby resulting in increased bulb yield [38]. This scenario could be attributed to the fact that elevated levels of nitrogen, phosphorus, and sulfur facilitated metabolic pathways, leading to the synthesis of nucleic acids, phospholipids, coenzymes, and chlorophyll. Consequently, these processes contributed to the increased bulb weight of garlic plants [39].

The total bulb yield of all garlic varieties in both types of soil was notably influenced by the application of varying rates of NPS. Additionally, as the rates of NPS increased, the total bulb yield of all garlic varieties also increased. However, surpassing the blanket recommendation for NPS resulted in a decrease in the overall bulb production of all garlic varieties (Table 9). Similarly, previous studies indicated that higher rates of blended NPS lead to increased bulb yield in garlic [12]. Furthermore, sulfur and nitrogen enhanced chlorophyll production and enzymatic activities, which promoted plant growth, development, and ultimately, high yields [39].

## 5 Conclusion

This research was conducted to assess the impact of four rates of NPS on the growth and yield of four varieties of garlic in a controlled greenhouse environment, to identify garlic varieties that grow well in the study area, and to determine the optimum rates of NPS. Results indicated a significant influence (*P* < 0.05) of four rates of NPS on the growth and yield of garlic varieties. The most effective fertilizer rate for Awada soil was determined to be 131.25-115-21.25 kg ha^−1^, while for Wondo Genet soil, the optimal rate was 78.75-69-12.75 kg ha^−1^. Among the garlic varieties tested, Tsedey 92 and Kuriftu exhibited the highest yields in Awada and Wondo Genet soils respectively, indicating that garlic varieties improved by the Debre Zeit Agricultural Research Centre displayed superior growth and yield characteristics compared to the local variety. Moreover, the study underscored the importance of supplementing NPS to enhance overall productivity.

## CRediT authorship contribution statement

**Eshet Lakew Tesfaye:** Writing – review & editing, Writing – original draft, Visualization, Validation, Supervision, Software, Resources, Project administration, Methodology, Investigation, Funding acquisition, Formal analysis, Data curation, Conceptualization. **T. Bayih:** Visualization, Validation, Supervision, Resources, Formal analysis, Data curation.

## Data availability

Data will be made available on request.

## Funding

This research received a grant from 10.13039/501100009698Hawassa University.

## Declaration of competing interest

The authors declare that there are no known competing financial interests or personal relationships that could have appeared to influence the work reported in this paper.
